# The Effect of Inflammation on Bone

**DOI:** 10.3389/fphys.2020.511799

**Published:** 2021-01-05

**Authors:** Scott Epsley, Samuel Tadros, Alexander Farid, Daniel Kargilis, Sameer Mehta, Chamith S. Rajapakse

**Affiliations:** ^1^Philadelphia 76ers, Philadelphia, PA, United States; ^2^Department of Radiology and Orthopaedic Surgery, University of Pennsylvania, Philadelphia, PA, United States; ^3^Orlando Magic, Orlando, FL, United States

**Keywords:** bone, inflammation, osteoblasts, osteoclasts, cytokines

## Abstract

Bone remodeling is the continual process to renew the adult skeleton through the sequential action of osteoblasts and osteoclasts. Nuclear factor RANK, an osteoclast receptor, and its ligand RANKL, expressed on the surface of osteoblasts, result in coordinated control of bone remodeling. Inflammation, a feature of illness and injury, plays a distinct role in skewing this process toward resorption. It does so via the interaction of inflammatory mediators and their related peptides with osteoblasts and osteoclasts, as well as other immune cells, to alter the expression of RANK and RANKL. Such chemical mediators include TNFα, glucocorticoids, histamine, bradykinin, PGE2, systemic RANKL from immune cells, and interleukins 1 and 6. Conditions, such as periodontal disease and alveolar bone erosion, aseptic prosthetic loosening, rheumatoid arthritis, and some sports related injuries are characterized by the result of this process. A thorough understanding of bone response to injury and disease, and ability to detect such biomarkers, as well as imaging to identify early structural and mechanical property changes in bone architecture, is important in improving management and outcomes of bone related pathology. While gut health and vitamin and mineral availability appear vitally important, nutraceuticals also have an impact on bone health. To date most pharmaceutical intervention targets inflammatory cytokines, although strategies to favorably alter inflammation induced bone pathology are currently limited. Further research is required in this field to advance early detection and treatments.

## Introduction

Inflammation is generally understood to promote resorption in bone (Boyce et al., 2005; Tanaka et al., 2005; Hardy and Cooper, 2009). A number of local and systemic mechanisms, particularly those involving inflammatory cytokines, have been elucidated (Adamopoulos, 2018). Inflammatory signaling pathways and chemical messengers that participate in bone remodeling will be discussed in depth, as well as a review of the OPG/RANKL system that regulates bone remodeling.

Much has been learned from studying the effects of periodontitis on alveolar bone loss in contributing to our understanding of immune and inflammatory mediated pathways in bone remodeling (Hienz et al., 2015), as has research into bone erosion secondary to rheumatoid arthritis (Wong et al., 2006). However, there is a paucity of information on the effects of local inflammation in the appendicular skeleton. A theoretical framework for the link between local inflammation and bone changes, termed here as the Outside-In and Inside-Out models, will be presented using an exertional leg pain model and drawing on what is already known about rheumatoid induced bone erosion.

Gastrointestinal mechanisms in inflammatory auto-immune disease that impact nutritional state and nutrient absorption have also been implicated. We will touch on the effects of calcium and vitamin D on bone health, as well as introduce the Gut-Bone Axis concept that has gained significant attention in recent years. Various nutraceuticals with evidence for impacting bone turnover will be explored.

Despite this knowledge, identification and targeted interventions for inflammatory induced bone resorption remain limited. The role of radiologic testing and blood chemistry markers in monitoring bone in inflammatory states will be reviewed in the context that an improved understanding of these mechanisms and early detection may lead to earlier intervention and improved patient outcomes. This paper seeks to raise clinician awareness and the need for further research to advance this area.

## Bone and Its Remodeling

Bone remodeling is a continuous process that renews the adult skeleton. It is distinct from bone modeling which is responsible for skeletal development, growth, and the shaping of bones, by the coordinated sequential action of osteoblasts and osteoclasts (Langdahl et al., 2016). The process is regulated both locally and systemically and is characterized by a resorption period lasting 30–40 days, and a formation period as long as 150 days (Eriksen, 2010). This cellular coordination is carried out by basic multicellular units (BMUs) (Khosla, 2001; Jilka, 2003) and forms resorption pits or lacunae (Everts et al., 2002; Eriksen, 2010). Bone lining cells then remove remaining collagen prior to new collagen matrix deposition and filling of the lacunae with new bone (Everts et al., 2002).

Bone remodeling is mediated by osteoclasts, osteoblasts, bone lining cells and osteocytes. Osteoclasts are large multiple nucleated cells that are responsible for breaking down bone tissue. They digest bone mineral by creating an acid compartment by acting as a proton pump and then release proteases, such as Tartrate-resistant Acid Phosphatase (TRAP) to degrade both inorganic and bone components, respectively (Boyle et al., 2003; Halleen et al., 1999; Owen and Reilly, 2018). Osteoclasts arise from hematopoeitc progenitors as osteoclast pre-cursors (Jilka, 2003) and are chemotactically attracted to resorption sites, where they fuse into mature osteoclasts (Pfeilschifter et al., 1989). Bone lining cells then remove any remaining debris via matrix metalloproteinases (MMP’s) (Everts et al., 2002). Various chemotactic agents including Parathyroid Hormone (PTH), TNFα, and Prostaglandin E2 (PGE2) upregulate RANKL expression which binds to its receptor RANK on osteoclast pre-cursors, leading to fusion and mature osteoclast formation (Roux and Orcel, 2000). RANKL is a Tumor Necrosis Factor (TNF) related ligand expressed on the surface membrane of osteoblasts that regulate their function (Roux and Orcel, 2000; Khosla, 2001). Osteoblasts are cells that work to synthesis bone. During bone formation, osteoblasts work as a group of connected cells within a functional unit called the osteon. Osteoblasts originate as mesenchymal stem cells (Jilka, 2003). Osteocytes are cells that originate from osteoblasts and become lodged in mineralized bone matrix. They participate in formation of bone, and the maintenance of matrix. Osteocyte apoptosis has been associated with bone fatigue, microcracks, and osteoclastic resorption indicative of early bone remodeling (Verborgt et al., 2000).

The attraction of pre-osteoclasts to a remodeling site is also chemotactically controlled by the osteoblast. Macrophage Colony Stimulating Factor (M-CSF) and Monocyte Chemoattractant Protein 1 (MCP 1) is secreted by osteoblasts in response to cytokines, such as TNFα and IL-1 and attracts osteoclast pre-cursors to the area (Graves et al., 1999). Various other important bone proteins include Osteoprotegerin (OPG) which binds to the RANK ligand (RANKL), blocking its interaction with RANK on the osteoclast pre-cursor cell, thereby inhibiting mature osteoclast formation (Roux and Orcel, 2000; Khosla, 2001; Boyce and Xing, 2008). Proteases released by resorbing osteoclasts activate transforming growth factor beta-1. (TGF-β1). TGF-β1 attracts osteoblasts and enhances proliferation and differentiation, proteoglycan synthesis, and type II collagen production. Thus, the process of resorption and formation is closely coupled to maintain bone homeostasis (Janssens et al., 2005).

## Inflammatory Factors That Participate in Bone Remodeling

Inflammatory cytokines, including interleukins (IL), IL-1, IL-6, and TNFα, have been found to have a significant effect on the bone remodeling process, mostly driving the system in the direction of resorption. Various neuropeptides have also been implicated.

Described below are some of the participants of inflammation signaling that also facilitate the bone remodeling processes:

### Cytokines

As well as stimulating M-CSF and MCP-1 to attract osteoclasts, TNFα and IL-1 stimulate the activity of mature osteoclasts, and attract other monocytes (Pfeilschifter et al., 1989). Macrophages between bone lining cells of the endosteum and periosteum have been shown to release TNFα (Chang et al., 2008). Furthermore, TNFα promotes systemic RANKL production by lymphocytes and endothelial cells (Boyce et al., 2005), while IL-1 acts on osteoblasts to induce Prostaglandin E2 (PGE2) synthesis, both indirectly inducing osteoclast formation. IL-1 also has an effect on RANKL expression that appears dependent on PGE2 (Miyaura et al., 2003; Tanaka et al., 2005). TNFα and prostaglandins play a pivotal role in osteoclast maturation. Osteoclast precursors require TNFα in the presence of small amounts of RANKL in order to differentiate into mature osteoclasts (Lam et al., 2000). Furthermore, when prostaglandin production is inhibited in cyclooxygenase-II (COX2) knockout mice, upregulation of Granulocyte Macrophage-Colony Stimulating Factor (GM-SF) occurs which inhibits osteoclast formation from pre-cursors (Tanaka et al., 2005). The use of a TNFα inhibitor (Etanercept), when combined with Methotrexate, were shown to reduce radiographic disease progression in those with rheumatoid arthritis despite high circulating inflammatory markers. This would imply that TNFα, independent of other systemic factors, has a significant effect on bone resorption, and that it may be possible to interrupt this process (Landewe et al., 2006).

IL-6 concentrations have been found to correlate with levels of joint erosion in rheumatoid arthritis sufferers, indicating a role in bone resorption (Kotake et al., 1996). Much like other inflammatory cytokines, it acts on osteoblasts and T-lymphocytes to increase RANKL production. In experimentally induced arthritis, IL-6 deficient mice show significantly less bone erosion (Wong et al., 2006). Much like IL-1, IL-6 also induces PGE2 production (Liu et al., 2005).

### Mast Cells

Mast cells may also contribute to the release of IL-6 and TNFα (Konttinen et al., 1996). They have further been implicated in studies investigating the effects of histamine 1 and histamine 2 receptor antagonists, in which they were shown to decrease the size of the osteoclast population (Dobigny and Saffar, 1997). This would suggest that mast cells are intricately linked with the osteoclastic bone resorption cascade.

### Neuropeptides

Neuropeptides have been implicated in bone resorption. Bradykinin, a vasoactive peptide with effects on vessel permeability and associated with inflammation, was found to stimulate the release of calcium from mice bones 24 h after administration. This process was inhibited by non-steroidal anti-inflammatories. Combined with the delayed response it is thought that bradykinin acts in bone resorption by mediating prostaglandin synthesis (Lerner et al., 1987). Other neuropeptides, such as Substance P and Vasoactive Intestinal Peptide (VIP) may also contribute to bone resorption (Konttinen et al., 1996).

## Signaling Pathways That Lead to Bone Loss

A relationship between inflammation and bone disease has been observed in a variety of clinical and laboratory settings, but the pathophysiology underlying this has yet to be fully appreciated. Diseases, such as chronic joint diseases, inflammatory bowel disease, lung inflammation, and renal diseases share many of the same mechanisms that can lead to bone loss, driven by immune signals that can tip the balance of bone homeostasis toward bone resorption (Hardy and Cooper, 2009). The bone remodeling cycle refers to this coordinated process, described earlier as osteoclasts removing bone at the microscopic level and osteoblasts replacing this matrix which ultimately re-mineralizes. The link between inflammation and the signaling pathways involved in this process will now be outlined.

### OPG/RANKL System

One of the major breakthroughs in understanding bone homeostasis at the molecular level was the discovery of the OPG/RANKL system. Osteoblasts express RANKL on their cell surface, the expression of which is upregulated in response to proinflammatory cytokines, glucocorticoids, estrogen deficiency and hyperparathyroidism (Hofbauer et al., 2000). RANKL then binds to the RANK receptor on the surface of osteoclasts and their precursors. Osteoprotegerin (OPG) is produced by osteoblasts and B lymphocytes and inhibits osteoclastogenesis. It acts as a decoy receptor, binding with RANKL to block its activation with RANK (Chen et al., 2018). Osteoclast precursors are cells that express a monocyte lineage marker (usually CD14, the M-CSF receptor, or CD11b) as well as RANK. The binding of RANKL to RANK induces a series of signal transduction pathways mediated through TNF receptor-associated factor 6 (TRAF6), which includes Nuclear Factor kappa-light-chain-enhancer of activated B cells (NF-kB) and Nuclear factor of activated T-cells (NFAT), that initiates the differentiation of the early osteoclast progenitor into a preosteoclast. Continued stimulation promotes the fusion of these preosteoclasts into the mature, multinucleated bone resorbing osteoclasts which can be recognized by any number of molecular markers (Weitzmann, 2013). Thus, balancing RANKL and OPG determines bone homeostasis, and if there will be net bone formation or resorption (Agrawal et al., 2011). Inflammatory cytokines like IL-1, TNF-α, and M-CSF have previously been shown to have associations with osteoclastic bone loss, by either promoting RANKL production by bone marrow stromal cells (osteoblast precursors) or mature osteoblasts (Hofbauer et al., 1999); by reducing OPG production (Weitzmann et al., 2002); or by promoting RANK on osteoclast precursors and thereby increasing their sensitivity to RANKL (Arai et al., 1999).

TNF-a has a particularly potent osteoclastic effect, likely due to the fact that RANKL is itself a TNF-superfamily member and functions through many of the same signal transduction pathways that TNF-a induces. TNF-a has been shown to act synergistically with IL-1 to upregulate RANKL expression on stromal cells and cause osteoclastogenesis (Wei et al., 2005). Against the background of inflammation, other cell types can supply RANKL in addition to stromal cells and osteoblasts. These include lymphocytes and fibroblasts, which constitute a large portion of the cells present in an inflamed synovium. The presence of this non-osteoblastic RANKL stimulates osteoclastogenesis independent of osteoblastic negative feedback, likely playing a role in the observed pathology. The recently identified T-helper cell 17 (Th17) subset of T-cells, which secrete the particularly osteoclastic cytokine IL-17, have been observed in inflammatory arthritis, and may explain the bone destruction that is commonly seen in that disease (Lundy et al., 2007).

Not all inflammatory cytokines demonstrate this effect. Some cytokines, such as IL-4, IFN-gamma, and TFG-β, have an inhibitory effect on osteoclastogenesis (Lorenzo et al., 2008). In the setting of bone disease however, the effect of these inhibitory cytokines is outweighed by those favoring osteoclastogenesis and the balance skews in favor of resorption. Another hypothesis is that osteoclastic cytokines can uncouple bone formation from bone resorption. Some studies have found that TNF-a can disrupt the differentiation of osteoblasts (Gilbert et al., 2000), and the presence of inflammatory cells may directly interrupt the signaling that couples these two processes, although the details of how this happens remains to be elucidated completely. A likely mechanism involves proteins synthesized by Wingless (Wnt) genes that mediate osteoblastogenesis. TNFα has been shown to upregulate production of Dickkopf-1 (DKK-1) which bind to and block Wnt receptors LPR5/6, suppressing osteoblast development. This system is integrated with the RANKL/OPG system whereby increased circulating levels of DKK-1 decreased levels of OPG, ultimately leading to bone resorption, and DKK-1 inhibition has been shown to increase OPG levels (Diarra et al., 2007; Baker-LePain et al., 2011).

### Glucocorticoid Signaling

It is also worth mentioning the role of glucocorticoids in bone diseases. Glucocorticoid excess has a negative impact on bone by uncoupling bone formation from resorption. Glucocorticoids downregulate a number of important signaling pathways in osteoblasts, especially the IGF-1 and WNT signaling pathways (Wang et al., 2008), and also decrease osteoblast proliferation and osteoblast specific protein production (Eijken et al., 2006). Glucocorticoid excess can also lead to inappropriate bone resorption due to decreases in OPG, although long term glucocorticoid use may actually inhibit osteoclast differentiation and thus decrease bone resorption (Weinstein et al., 2002), the dominant effects are thought to be through decreased bone deposition and bone quality (Chiodini et al., 2016). Dexamethasone has been shown to interrupt NF-kB signaling, inhibiting osteoclast maturation, leading to osteopetrosis (Unlap and Jope, 1997).

The role of endogenous glucocorticoids in bone disease is evident from patients with Cushing’s syndrome and other pathologies characterized by increased cortisol release who demonstrate a higher prevalence of vertebral fractures. Trabecular bone appears to be more sensitive to the effects of endogenous glucocorticoids than cortical bone (Wetzsteon et al., 2009), with changes in cortical bone equivocal (Chiodini et al., 2016).

While chronic exogenous glucocorticoid therapy is well-known to have deleterious effects on bone health as evidenced by 30–50% increased fracture risk (Canalis et al., 2007), chronic inflammatory disease itself may detrimentally impact bone, with Chronic Obstructive Pulmonary Disease associated with a higher risk of osteoporosis independent of glucocorticoid use (Chen et al., 2015). In fact, treatment of chronic inflammatory disease with glucocorticoids may have a beneficial effect on bone in some cases. Bone mineral density was not correlated to the cumulative dose of corticosteroid therapy in a study of children with Inflammatory Bowel Disease but was inversely related to the cytokine IL-6. This would suggest more research is required to understand the exact way in which glucocorticoids affect bone modeling (Paganelli et al., 2007).

## Immunologically Driven Bone Loss Pathologies

Several pathological models have been utilized to better understand the local tissue inflammatory mediated bone response. Periodontitis, rheumatoid arthritis, and aseptic prosthesis loosening will be discussed below:

### Periodontitis

Periodontitis is a dysbiotic disease in which the oral microbiota become dysregulated and lead to an increased risk of systemic inflammatory diseases, such as Rheumatoid arthritis (RA) (Hajishengallis, 2015). There has been increasing interest in understanding the pathophysiology of periodontitis due to its rising prevalence and its potential use as a model to study bone resorption (Hienz et al., 2015). While it is known that both the microbial infection and inflammatory immune responses play a role in bone resorption due to periodontitis, the specific mechanism underlying this has not been clearly defined (Nanci and Bosshardt, 2006; Abusleme et al., 2013). One obstacle in identifying the precise role of the various factors involved is the overlapping nature of their effects. For example, many cytokines have multiple roles throughout the body. The field of osteoimmunology has been helpful in defining the complex interactions between bone, surrounding tissue, and the resulting inflammatory response (Hienz et al., 2015). Bacterial infection of the tissue surrounding and supporting the tooth is also known to trigger an inflammatory response, and it is worth noting that this response is dependent on the specific tissue type and its function (Nanci and Bosshardt, 2006).

Helper T-cells have been implicated in the immunopathogenesis of bone resorption. These cells are generally classified into two subsets; Th1 cells (involved in cell-mediated response to intracellular infection), and Th2 cells (involved in response to extracellular infection). RANKL expression on the surface of Th1 cells has been associated with bone loss in periodontitis (Taubman et al., 2005). Suppression of Th1 cells in rat models have resulted in a decrease in bone resorption and RANKL expression (Valverde et al., 2004). Interestingly, RANKL-expressing B lymphocytes have been shown to increase bone resorption even in the absence of RANKL-expressing helper T cells (Taubman et al., 2005). Conversely, T cells promote OPG production by B lymphocytes, and thus this interaction between lymphocytes is critical for bone homeostasis (Li et al., 2007).

### Rheumatoid Arthritis (RA)

Rheumatoid arthritis (RA) is another systemic inflammatory disease primarily caused by an excess of pro-inflammatory cytokines resulting in inappropriate immune response. The disease is characterized by inflammation of the synovial membrane, surrounding cartilage, and bone (McInnes and Schett, 2007), and affects approximately 1% of people worldwide (Gabriel, 2001).

An autoimmune inflammatory response can last for years before bone and cartilage loss accelerates (Dekkers et al., 2016; Weyand and Goronzy, 2017). Synovial macrophages in RA produce inflammatory cytokines previously described to induce bone resorption including TNF-a, IL-1, and IL-6 (Li et al., 2012). Furthermore, RANKL is expressed in RA synovial fibroblasts (Kim et al., 2007) promoting differentiation of synovial macrophages into osteoclasts (Takayanagi et al., 1997).

The development of the disease has a genetic component, as evidenced by concordance rates of around 15% for monozygotic twins and 3% for dizygotic twins (Silman et al., 1993). It has been proposed that genetic factors may account for as much as 60% of the liability to the disease (MacGregor et al., 2000). There are also significant associations with immunological regulatory genes as demonstrated by genome-wide association study (GWAS) analysis, providing further evidence for a genetic predisposition to developing RA. It has been suggested that the gastrointestinal microbiota plays a role in onset of RA as well (Scher et al., 2010).

While the mechanism of immune response and eventually bone loss are still to be well elucidated it is likely that glucocorticoids play a significant role. As previously stated, glucocorticoids stimulate the process of bone resorption, increase the expression of RANK, and decrease the expression of OPG (Canalis and Delany, 2002). Treatment with glucocorticoids have played a role in the clinical management of patients with RA for some time due to symptomatic relief, however, adverse effects have led to their use in low dosages as a “bridge therapy” to anti-rheumatic drugs (Van Gestel et al., 1995). Also of interest is the relationship between stress and bone resorption for patients with chronic inflammatory diseases. Continual stress is consistently associated with increased inflammation and bone resorption for patients with RA (Straub et al., 2005).

### Aseptic Prosthesis Loosening

Several groups have found that it is possible for macrophages themselves to induce a low-grade kind of bone resorption. Aseptic loosening of the prosthetic is one of the major reasons for joint replacement failure. Inflammatory cells, such as foreign-body macrophage polykaryons are known to aggregate at the border of the native bone and cemented prosthesis and can be characterized histologically. The resorptive pits that these cells create can be seen microscopically (Athanasou et al., 1991). Macrophages found in pseudo-synovial tissue surrounding joint replacements have been shown to be accompanied by T-cells and demonstrated high levels of inflammatory cytokines (Perry et al., 1995). RANKL has also been identified in this tissue providing further impetus for osteoclastogenesis and resorption (Horiki et al., 2004).

That macrophages can engage in bone resorptive behavior challenged a previous paradigm that only osteoclasts could resorb bone, although the character of macrophage resorption was noticeably different. Bone resorption done by macrophages is typically low-grade, with fewer pits and smaller diameter. The major implication of this is that anywhere in the body that may excite macrophages, such as areas with a lot of cellular death products, or bacterial and foreign particulate matter, may be prone to low-grade bone resorption. This has to be considered clinically, especially with regard to joint replacements, because this resorption could lead to loosening of the prosthesis (Horowitz and Purdon, 1995).

## Outside in and Inside Out Models

While there is an established body of literature linking chronic and systemic inflammatory responses to bone resorption, there is paucity with respect to local inflammation. The pathophysiology of conditions, such as medial tibial stress syndrome (MTSS) has been theorized to comprise either a periostitis secondary to fascial traction (Michael and Holder, 1985; Bouché and Johnson, 2007; Stickley et al., 2009) or a distinctly bony etiology characterized by decreased regional bone density and high resolution CT abnormalities (Magnusson et al., 2001; Gaeta et al., 2006).

Histology from biopsies of the painful regions of MTSS patients that failed conservative treatment demonstrated active osteoblasts, inflammatory changes in the crural fascia, and in one case inflammatory infiltrate into the lymphatics of the periosteum (Johnell et al., 1982). Furthermore, inflammatory cells were noted in the fascia of those suffering from chronic exertional compartment syndrome (Barbour et al., 2004). Periosteal osteoblasts have been shown to be sensitive to physiological traction strains, responding with an increased production of PGE2 (Forwood et al., 1998; Jones et al., 1991). Given the established local inflammatory environment, increased PGE2 production from fascial traction, and previously discussed effects of both (Tanaka et al., 2005), it is not inconceivable that a bony response could follow. This model, in which the presence of an inflammatory infiltrate in the local environment inhibits the formation of new bone, has been described as the “outside-in” model. Although induced by an acute local inflammatory response, this is analogous to bone resorption secondary to autoimmune mediated synovial inflammation in RA (Li et al., 2012).

In contradiction to this theory biopsies of 6 symptomatic MTSS patients, only noted one associated bone remodeling front from 3/6 tibiae that exhibited microcracks. However, that signifies a remodeling response in one third of samples exhibiting bone micro-trauma (Winters et al., 2019).

**FIGURE 1:**
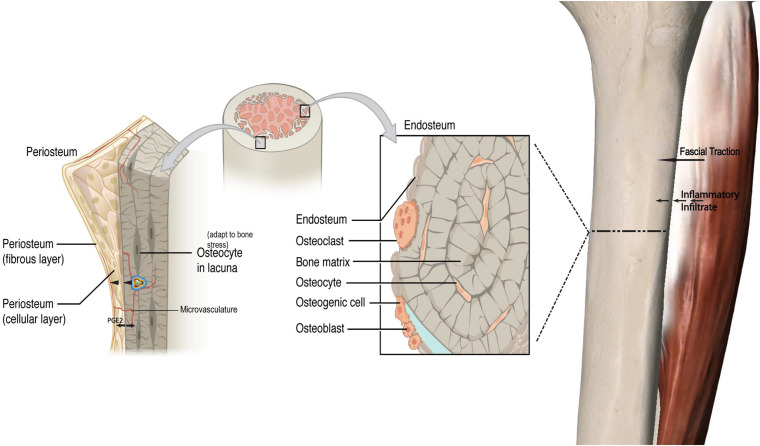
Fascial-bone stress model. A visual framework depicting a proposed pathway of an outside-in model for bone remodeling with a local inflammatory response via fascial traction and resultant bony response with impact on periosteal osteoblasts and increased production of PGE2. Similar to the outside-in model with cascade of inflammatory processes coming from fascial layers into periosteum and endocortex, a proposed inside-out mechanism is illustrated. This load induced bone stress response along with increased vasculature and inflammatory cytokines originating from the endocortex drives periosteal change in addition to crosstalk between the endocortical and periosteal layers. *PGE2* Prostaglandin E2. The cell in cocentric multicolor lines represent an inflammatory cytokine (not to scale). Adapted from https://openstax.org/books/anatomy-and-physiology/pages/6-3-bone-structure.

While an outside-in model for bone remodeling is proposed, this could be extended to an “inside-out” model as well. Transcortical vessels, capillaries in long bones linking the marrow and traversing vertically and horizontally to connect to the periosteum have recently been described. Proliferation of these vessels has been observed within weeks in chronic arthritic bone inflammation (Grüneboom et al., 2019). These vessels express arterial or venous markers and transport neutrophils. Given that periosteal osteoblasts make direct contact with the bone cortex (Squier et al., 1990) and have cytoplasmic projections into the osteoid (Ellender et al., 1988), and that there is now an established vascular connection, this may be a pathway by which chemotactic agents and even monocytes induce periosteal changes secondary to an endocortical response. In keeping with this, mechanical loading has been demonstrated to induce significant and rapid PGE2 release in the metaphysis (Thorsen et al., 1996). This type of response may be represented in non-invasive modalities, such as MRI’s where both endocortical and periosteal signal co-exist to represent a bone stress response (Batt et al., 1998). Increased microvasculature density has been correlated with local histological inflammatory changes in facets joints of Ankylosing Spondylitis patients who also demonstrate bone marrow edema, strengthening the argument for a link between endocortical and superficial cortical bone (Appel et al., 2006a). Figure 1 illustrates the fascial-bone stress model.

## Nutrition, Microbiota, and Bone Health

In addition to inflammatory processes, nutritional status is also important for bone health. Inflammation and nutritional status are not mutually exclusive. Chronic inflammatory conditions of the gastrointestinal tract are frequently associated with poor nutritional status, largely having to do with a reduction in caloric intake and difficulty in absorbing nutrients important to bone metabolism (Corazza et al., 2005). Moreover, the gut microbiota plays a role in systemic inflammation via the immune system (D’Amelio and Sassi, 2018), and meal ingestion can influence bone remodeling (Villa et al., 2017).

### Calcium

Coelic disease is a chronic inflammatory disease of the gastrointestinal tract that can impact the absorption of calcium and vitamin D (Corazza et al., 2005). These nutrients are crucial in maintaining adequate mineralization of bone, and the body responds to deficiency by increasing parathyroid hormone (PTH) secretion. PTH receptors expressed on osteoblasts provide increased signaling that results in increased RANKL expression. This elevated RANKL increases osteoclastogenesis and leads to bone resorption in order to increase serum calcium levels to compensate for the deficiency. However, if calcium absorption is limited because of impaired gut absorption this cycle will continue. Bone will continue to be resorbed and remineralization will be impaired. As such a lack of calcium and vitamin D can adversely affect bone mineralization independent of the bone remodeling cycle.

### Vitamin D

Vitamin D has an additional physiologic role in that it can also modulate the immune system. Vitamin D has demonstrated an anti-inflammatory role in diseases, such as kidney disease, rheumatoid arthritis, and inflammatory bowel disease (Zehnder et al., 2008). Low levels of vitamin D are correlated with a greater degree of inflammation in these conditions. Immune cells have the capacity to convert the precursor to active vitamin D, 25-hydroxyvitamin D, to the active form, 1,25-hydroxyvitamin D (Adams and Hewison, 2008). This active form of vitamin D functions as a steroid hormone and binds to the nuclear vitamin D receptor (VDR), of which there are particularly high levels in macrophages, dendritic cells, and lymphocytes (Mousa et al., 2016). This VDR suppresses the proliferation of lymphocytes and downregulates pro-inflammatory cytokines like TNF-α, IL-1, IL-6, and IL-8, while upregulating IL-10 which is an anti-inflammatory cytokine. VDR also seems to promote the differentiation of monocytes into macrophages and inhibits their ability to secrete inflammatory cytokines and express MHC-II molecules at their surface, thereby reducing their inflammatory profile (Guillot et al., 2010). VDR also appears to downregulate NF-kB, an important proinflammatory transcription factor (Harant et al., 1997). Despite this, supplementation with vitamin D has not been shown to be effective in treating inflammatory diseases in a clinical setting.

It is clear that poor nutrient intake as the result of an inflammatory disease may affect the bone remodeling process as we have just detailed. Other conditions characterized by poor nutrient intake without an inflammatory environment, such as anorexia nervosa display a similar uncoupling of bone formation and resorption (Soyka et al., 1999). The reason for this is not well understood, but at least part of this bone remodeling is governed by central inputs (Karsenty, 2006).

### Gut Bone Axis

The gut microbiota (GM) is defined as the whole system of symbiotic and pathogenic microorganisms inhabiting our intestines (Quach and Britton, 2017; Villa et al., 2017; D’Amelio and Sassi, 2018). The GM has a complex relationship with its host aiding in digestion, battling pathogens, and maturation of the immune system in the first years of life. These constant interactions between GM and the host contribute to the variation of gut and systemic immunity throughout life (D’Amelio and Sassi, 2018). However, disturbances in immune homeostasis can serve as a contributing factor for chronic non-communicable human diseases (NCDs) like allergies, asthma, some autoimmune, cardiovascular and metabolic diseases, and neurodegenerative disorders (D’Amelio and Sassi, 2018). Alterations in GM and host interaction has been associated with a possible cause of immune disruption and increased inflammation associated with several NCDs (Peterson et al., 2015).

The GM-Bone axis is defined as the effect of the GM, or the molecules they synthesize, on bone health (Villa et al., 2017). A symbiotic interplay between immune and bone cells, leads the GM to have a central role in maintaining bone health along with influencing bone turnover and density (Mori et al., 2015). GM can improve bone health by increasing calcium absorption and modulating the production of gut serotonin, a molecule that interacts with bone cells and has been suggested to act as a bone mass regulator (D’Amelio and Sassi, 2018). Manipulation of GM with changes in dietary habits, consumption of antibiotics, and probiotic use may positively influence bone health.

Similarly, glucagon-like peptide-1 (GLP-1) is a peptide hormone secreted from entero-endocrine L-cells post meal ingestion (Nissen et al., 2019). In a recent study on healthy, young male and female human subjects, CTX along with other bone markers were analyzed via blood sampling (Nissen et al., 2019). It concluded GLP-1 plays a role in the gut-bone axis since GLP-1 has an inhibitory effect on the bone resorption marker CTX. GLP-1 is known primarily as an insulinotropic hormone and together with glucose dependent insulinotropic polypeptide (GIP), both are responsible for increased insulin secretion after oral ingestion of glucose (Nissen et al., 2019). Bone remodeling can vary daily with a decrease in bone resorption postprandially, whereas the opposite is true when fasting. Thus, GLP-1 contributes to the regulation of bone turnover as part of the gut-bone axis. In summary, the gut-bone axis has become an emerging topic and GM plays a multi-faceted role in bone turnover, modulating the immune system, controlling inflammation, interacting with key hormones, and ensuring appropriate absorption of calcium and vitamin D levels (Quach and Britton, 2017; Villa et al., 2017).

### MicroRNA and Bone Remodeling

**FIGURE 2:**
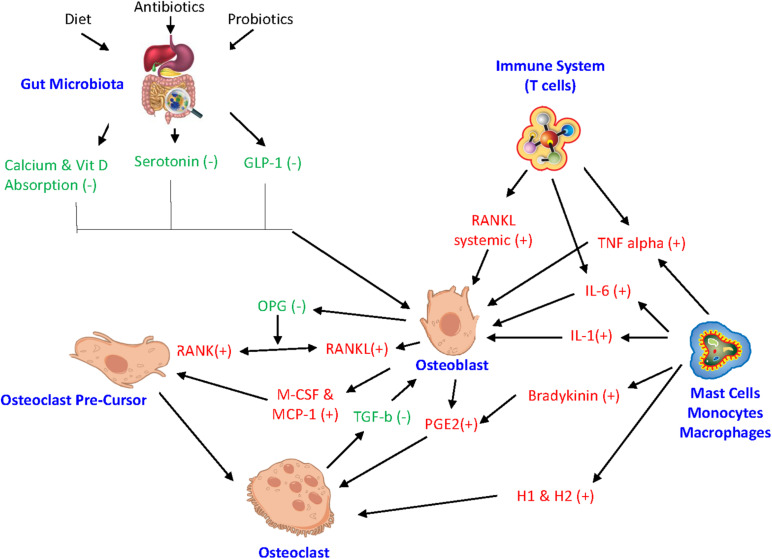
Integrated inflammatory model. The complex link between gut microbiota, hormonal pathways, immune system, and bone turnover. *GLP-1* glucagon-like peptide, *miRNA* microRNA, *IL-6* interleukin-6, *IL-1* interleukin-1, *RANKL* receptor activator of NF-kB ligand, *RANK* receptor activator of nuclear factor-kB, *OPG* Osteoprotegrin, TNF *alpha* tumor necrosis factor-alpha, *M-CSF* macrophage colony stimulating factor, *MCP-1* monocyte chemoattractant protein 1, *H1* histamine 1, *H2* histamine 2, *PGE2* Prostaglandin E2. (+) drive osteoclastogenesis or resorption. (−) inhibit resorption.

In addition, the activity of bone cells is controlled by a variety of factors, such as their own intracellular molecular processes. Any impairment of these intracellular processes can affect bone homeostasis. MicroRNAs (miRNAs) are a type of RNA that regulate biological processes, like posttranscriptional intracellular protein expression (Liu et al., 2019; Zhao et al., 2019). Studies have found that miRNA play a key role in mediating osteoblast, osteoclast, and osteocyte activity while any miRNA deregulation can result in impaired bone remodeling (Sugatani and Hruska, 2009; Zhao et al., 2019). Also, the proinflammatory cytokine TNF-a is involved in the pathogenesis of chronic inflammatory diseases and ultimately plays a role in osteoclastic activity (Liu et al., 2019). Suppression of certain miRNAs supports the mechanism to restrain TNF-a induced bone resorption. Relocating to outside the cell, miRNA can also control exosomes serving as intercellular signals to facilitate cell to cell crosstalk among bone cells (Liu et al., 2019). Figure 2 illustrates the integrated inflammatory model.

## Endocrine Factors in Inflammation and Bone Health

Another important factor to consider in bone health is the role of hormonal signals. Endocrine factors, such as leptin signal in the central nervous system and mediate their effects on bone through the sympathetic nervous system. Mature osteoblasts and chondrocytes both have leptin receptors on their cell surface, suggesting leptin may be directly affecting these cells, potentially through activity of Fibroblast growth factor 23 (FGF-23) (Upadhyay et al., 2015). However, it is more likely that leptin’s effects on bone mass are indirect. One potential mechanism is that the ventromedial hypothalamus (VMH) activates local noradrenergic signaling in osteoblasts in response to leptin, stimulating them to form bone (Takeda et al., 2002). Leptin also works by downregulating serotonin and inhibiting serotonergic receptors in the VMH. Serotonin has the opposite effect of leptin and inhibits osteoblasts, thus decreasing bone growth (Yadav et al., 2009). Leptin is involved in the regulatory networks of several important endocrine hormones, such as cortisol, thyroid and parathyroid hormone, IGF-1 and growth hormone, and estrogen, all of which can impact the bone remodeling cycle. Therefore, leptin secreted from the central nervous system could affect bone directly or indirectly through a wide range of secondary endocrine mediators (Khan et al., 2012).

In humans it has been demonstrated that estrogen deficiency affects the number of bone cells, and bone turnover partially through its effect on the immune system. During estrogen deficiency, T cells increase their production of pro-inflammatory and pro-osteoclastogenic cytokines, TNF-α and RANKL (D’Amelio and Sassi, 2018). Estrogen therapy may also help prevent bone loss in post-menopausal women by altering the balance between IL-1 and its receptor agonist (Rogers and Eastell, 1998), providing further evidence for a link between the endocrine system and cytokine mechanisms of bone remodeling.

## The Role of Immobility on Bone Health

Another mechanism affecting bone health that remains poorly understood is immobility. Several groups have shown that bedridden patients display poorer bone health and are at an increased risk of fracture (Eimori et al., 2016). It has been speculated that osteocytes, embedded within the bone matrix, may be involved in mechanosensing. These cells likely work by modulating signaling, such as the wnt pathway which involves both bone formation and resorption, making them crucial for coupling these processes (Bonewald and Johnson, 2008). One study demonstrated that osteoblasts displaying estrogen receptor alpha, whose expression is upregulated in the presence of estrogen, responded to load via wnt pathway gene upregulation. They concluded that estrogen decline in both men and women may in part lead to failure to maintain appropriate bone mass via this decreased sensitivity to load (Armstrong et al., 2007). Immobility also typically restricts time spent outdoors thus limiting exposure to sunlight, decreasing vitamin D synthesis in the skin. Therefore, immobility can lead to decreased vitamin D levels which can negatively affect the mineralization of bone (Cooper and Gittoes, 2008). Furthermore, in rheumatoid arthritis immobility may compound bone loss due to inflammatory disease activity (Haugeberg et al., 2002). It would appear, however, that increasing mobility has the propensity to reverse these changes, with strength training demonstrating increased levels of osteocalcin and alkaline phosphatase, markers of bone deposition, as well as improved bone mineral density (Menkes et al., 1993).

## Interventions

Treatment for inflammatory disease affecting bone has progressed to specifically address inflammatory cytokines and their pathways in various different ways. Infliximab, a TNF-α antibody that blocks TNF-α receptor interactions has shown promise in the treatment of rheumatoid arthritis, as has Etanercept, an agent that binds with TNF-α preventing from interacting with its receptor. While successful in reducing joint erosion they are not without adverse effects and should be avoided in the presence of chronic infection and heart failure (Scott and Kingsley, 2006). Estrogen therapy may have an indirect effect on IL-1 and thus its negative influence on bone, by altering the balance between IL-1 and its receptor agonist (Rogers and Eastell, 1998). Gene therapy, specifically aimed at upregulating OPG, showed early promise in decreasing inflammation and bone resorption in aseptic prosthesis loosening (Wooley and Schwarz, 2004). More recently the influence of MMP inhibitor alpha-2-macroglobulin has been shown to decrease IL-1 beta and TNF-α resulting in lower osteoarthritis scores, although the direct influence on bone resorption is yet to be studied (Wang et al., 2014).

Monoclonal antibodies are now playing a role in management also. Rituximab is a monoclonal antibody that depletes B cell population by targeting their CD20 surface antigen, showing up to 20% improvement in RA symptoms (Edwards et al., 2004), while Tocilizumab, an anti-IL-6 monoclonal antibody demonstrated a decrease in bone degradation markers when combined with methotrexate (Garnero et al., 2010).

As previously stated, nutrition has an impact on bone health, and the influence of nutraceuticals should not go unmentioned. Green tea polyphenol supplementation, of which epigallocatechins are the active component, inhibited TNF-α and suppressed osteoclast activity (Shen et al., 2011), inhibited IL-6 expression and downregulated bone resorption (Wu et al., 2018), while 500 mg daily when combined with Tai Chi shifted the ratio of bone formation to resorption in a net positive direction (Shen et al., 2012). Curcumin has also been shown to inhibit RANKL induced NF-kB osteoclastogenesis *in vitro* in a dose dependent manner (Bharti et al., 2004).

## Contemporary Imaging in Inflammation and Bone Health

Lastly, we will discuss imaging and blood based methods to monitor inflammation and its effect on bone health that are currently in use today.

### Micro-Computed Tomography

Micro-computed tomography (uCT) has been used extensively to measure bone resorption in inflammation studies on animals (Lopes et al., 2018; Kuraji et al., 2018; Yoshihara-Hirata et al., 2018). uCT has been used to confirm that bone resorption was positively associated with levels of inflammation following administration of a 5-Lipoxygenase (5-LO) inhibitor; 5-LO has been implicated in inflammatory processes (Lopes et al., 2018). uCT has been used to confirm similar findings following administration of theaflavins, which are thought to reduce inflammation and subsequently reduce bone resorption (Kuraji et al., 2018). uCT used in a study on anti-HMGB1 neutralizing antibody and its effect on inflammation and bone resorption also demonstrated a positive correlation between levels of inflammation and bone resorption (Yoshihara-Hirata et al., 2018). It should be acknowledged that uCT administers a dose of ionizing radiation too aggressive and damaging for regular clinical use (Willekens et al., 2010).

### Magnetic Resonance Imaging

Magnetic resonance imaging (MRI) is a safer and ubiquitous imaging option in a clinical setting. Several studies have determined its efficacy in measuring bone resorption in response to inflammation. One study compared bone marrow edema (BME)—inflammation of bone marrow—seen on MR images to levels of inflammation and BMD in patients with ankylosing spondylitis (AS), a chronic systemic inflammatory disease (Wang et al., 2017). From this study, it was determined that MRI-based imaging was effective in showing high BME in areas of low BMD and high inflammation. However, in another study on similar patients the amount of BME on MRI was only well-correlated to mononuclear cell infiltrates and other histopathologic measures of inflammation when high levels of inflammation were present. These authors concluded that signal identified on T2 weighted MRI imaging likely represents water as a correlate of BME and inflammation, but not inflammation itself (Appel et al., 2006b).

### Positron Emission Tomography

Positron Emission Tomography (PET) is an effective method of detecting inflammation markers and visualizing the biological process of inflammation in a specific and sensitive fashion (Wu et al., 2013). PET tracers were able to more sensitively detect inflammation in the gastrointestinal tract, when compared to MRI (Dmochowska et al., 2019). In a study designed to investigate the bone metabolism and inflammatory characteristics of chronic non-bacterial osteomyelitis (CNO), fluorodeoxyglucose (FDG)-PET was utilized to locate regions of inflammation, even where symptoms of CNO were not present (Ata et al., 2017). PET could theoretically be used to help accurately and visually trace the inflammation process and its interaction with bone resorption.

### Ultrashort Echo Time MRI

Ultrashort echo time (UTE) MRI is another imaging technique utilized regularly today. This method allows for unprecedented imaging of microstructure and composition of cortical bone. More specifically, it can more accurately discern between different regions of bone, such as bone bound to the collagen matrix vs. perforations within the bone cortex vs. the water content of the bone (Siriwanarangsun et al., 2016). While it may be unable to detect inflammation directly, it can depict how the bone is being affected as a result of inflammation. Use of UTE-MRI in determining the interplay between inflammation and bone resorption has not yet been extensively studied. However, due to its uniquely detailed imaging of cortical bone, UTE-MRI may become an important tool in the future in determining the mechanism of remodeling bone.

## Blood Biomarkers

Blood chemistries focusing on inflammatory biomarkers have also demonstrated clinical utility. For example, it has been shown that higher levels of C-reactive protein (CRP), a marker of inflammation, is associated with lower bone mineral density (BMD), suggesting that bone resorption is increased in the inflammatory setting (Koh et al., 2005; Lim et al., 2016). Similarly, another study measured blood levels of various inflammatory markers—CRP, interleukin (IL)-1β, and IL-6, among others—with BMD and bone mineral content (BMC) over 6 and 12 months in postmenopausal women. It found that these markers accounted for 1.1–6.1% of variation in bone loss amongst their subjects, and suggests that altering inflammatory markers may lead to decreased bone loss (Gertz et al., 2010).

## Conclusion

It is clear that inflammation drives bone toward a resorption state. Osteoblasts have been highlighted as a central player, responding to inflammatory cytokines including TNFα and IL-1 by releasing MCS-F and MCP 1 to attract osteoclast pre-cursors. Systemic inflammation may also contribute, both chemotactically and via decreased mobility. Inflammatory mediators, such as TNFα stimulate RANKL production by lymphocytes and endothelial cells, and IL-1 and IL-6 induces PGE2 production by osteoblasts. Both mechanisms indirectly induce osteoclast formation and lead to bone resorption. Similarly, immune mediated responses via helper T cells and B lymphocytes appear to increase resorption. The discovery that macrophages play a role in bone resorption in aseptic prosthesis loosening has challenged the notion that only osteoclasts resorb bone. The effect of nutrition on bone status, specifically vitamin D and calcium is well-documented, however, the gut microbiota is less frequently considered but may be equally as important in both improving gut absorption and minimizing immune mediated systemic responses. The interplay between periosteal and endosteal bone remodeling may in part be explained by local inflammatory mediators, such as PGE2, IL-1, and IL-6 acting bi-directionally through periosteal, endosteal and synovial cell surface contact, and the recently described microcirculation. uCT and UTE MRI are emerging imaging modalities that provide for improved monitoring and identification of bone resorption, while blood monitoring of inflammatory markers may give measures of inflammation which have been shown to contribute directly to bone loss.

Despite well-described pathways for the effect of inflammation on bone, interventions to directly impact these pathways remain limited. While cytokine inhibitors appear to uncouple the relationship between inflammation and bone erosion, glucocorticoids appear to have differing effects in different populations. Nutraceuticals may also play a role in influencing inflammation driven resorption. More research is needed in these areas in order to develop interventions that protect bone in the presence of inflammation.

Limitations of this paper include an original, theoretical framework around specific players and pathways of inflammation involved in bone remodeling. These models uncover mechanisms involved with achieving bone homeostasis and identify potential actionable areas. Future research is warranted in humans to bring understanding of methodology to clinical practice and to determine if specific radiologic testing and blood biomarkers are key players in monitoring inflammation, directly and indirectly. However, these limitations also serve as strengths since there is a paucity of evidence on impact of local inflammation in the appendicular skeleton.

Finally, by understanding the effect of local and systemic inflammation on bone remodeling and the precise mechanisms by which this occurs, research into interventions can be better directed at minimizing inflammatory mediated bone resorption, thereby improving outcomes and hastening recovery from injury.

## Author Contributions

All authors contributed substantially to the drafting and editing of the manuscript.

## Conflict of Interest

SE was employed by Philadelphia 76ers. SM was employed by Orlando Magic. The remaining authors declare that the research was conducted in the absence of any commercial or financial relationships that could be construed as a potential conflict of interest.

## Abbreviations

RANK: receptor activator of nuclear factor-kappa B
RANKL: RANK ligand
TNF α: tumor necrosis factor alpha
PGE2: prostaglandin E2
OPG: osteoprotegerin
M-CSF: macrophage colony-stimulating factor
MMPs: matrix metalloproteinases
IL: interleukin
MCP 1: monocyte chemoattractant protein 1
COX2: cyclooxygenase-II
GM-SF: granulocyte macrophage-colony stimulating factor
VIP: vasoactive intestinal peptide
MRI: magnetic resonance imaging
uCT: micro-computed tomography
UTE MRI: ultrashort echo time MRI
PET: positron emission tomography
TRAF6: TNF receptor-associated factor 6
NF-kB: nuclear factor kappa-light-chain-enhancer of activated B cells
NFAT: nuclear factor of activated T-cells
Th17: T-helper cell 17
DKK1: dickkopf WNT signaling pathway inhibitor 1
IFN-gamma: interferon gamma
TGB-beta: transforming growth factor beta
IGF-1: insulin-like growth factor 1
AS: ankylosing spondylitis
MTSS: medial tibial stress syndrome
RA: rheumatoid arthritis
BME: bone marrow edema
VDR: vitamin D receptor
GM: gut microbiota
NCDs: non-communicable human diseases
GLP-1: glucagon-like peptide-1
CTX: C-terminal telopeptide
GIP: glucose dependent insulinotropic polypeptide
miRNAs: Micro Ribonucleic Acids
VMH: ventromedial hypothalamus
CNO: chronic non-bacterial osteomyelitis
FDG-PET: fluorodeoxyglucose-positron emission tomography
CRP: C-reactive protein
BMD: bone mineral density.
